# Identification of a Wee1–Like Kinase Gene Essential for Procyclic *Trypanosoma brucei* Survival

**DOI:** 10.1371/journal.pone.0079364

**Published:** 2013-11-05

**Authors:** Natalia Y. Boynak, Federico Rojas, Cecilia D’Alessio, Salomé C. Vilchez Larrea, Vanina Rodriguez, Pablo D. Ghiringhelli, María T. Téllez-Iñón

**Affiliations:** 1 Instituto de Investigaciones en Ingeniería Genética y Biología Molecular “Dr. Héctor N. Torres”, (INGEBI-CONICET), Buenos Aires, Argentina; 2 Laboratory of Glycobiology, Fundación Instituto Leloir and Instituto de Investigaciones Bioquímicas de Buenos Aires-CONICET, Buenos Aires, Argentina; 3 Department of Science and Technology, Universidad Nacional de Quilmes, Buenos Aires, Argentina; Instituto Butantan, Laboratório Especial de Toxinologia Aplicada, Brazil

## Abstract

Regulation of eukaryotic cell cycle progression requires sequential activation and inactivation of cyclin-dependent kinases (CDKs). Activation of the cyclin B-cdc2 kinase complex is a pivotal step in mitotic initiation and the tyrosine kinase Wee1 is a key regulator of cell cycle sequence during G2/M transition and inhibits mitotic entry by phosphorylating the inhibitory tyrosine 15 on the cdc2 M-phase-inducing kinase. Wee1 degradation is essential for the exit from the G2 phase. In trypanosomatids, little is known about the genes that regulate cyclin B-cdc2 complexes at the G2/M transition of their cell cycle. Although canonical tyrosine kinases are absent in the genome of trypanosomatids, phosphorylation on protein tyrosine residues has been reported in *Trypanosoma brucei.* Here, we characterized a Wee1-like protein kinase gene from *T. brucei*. Expression of TbWee1 in a *Schizosaccharomyces pombe* strain null for Wee1 inhibited cell division and caused cell elongation. This demonstrates the lengthening of G2, which provided cells with extra time to grow before dividing. The Wee1-like protein kinase was expressed in the procyclic and bloodstream proliferative slender forms of *T. brucei and the* role of Wee1 in cell cycle progression was analyzed by generating RNA interference cell lines. In the procyclic form of *T. brucei*, the knock-down of TbWee1 expression by RNAi led to inhibition of parasite growth. Abnormal phenotypes showing an increase in the percentage of cells with 1N0K, 0N1K and 2N1K were observed in these RNAi cell lines. Using parasites with a synchronized cell cycle, we demonstrated that TbWee1 is linked to the G2/M phase. We also showed that TbWee1 is an essential gene necessary for proper cell cycle progression and parasite growth in *T. brucei*. Our results provide evidence for the existence of a functional Wee1 in *T. brucei* with a potential role in cell division at G2/M.

## Introduction

Regulatory pathways controlling the eukaryotic cell cycle have been very well studied in yeast and higher eukaryotic cells and have been shown to involve an intricate net of regulatory proteins such as cyclins, cyclin-dependent kinases (CDKs) and CDK inhibitors (CKIs) [1]. The activity of CDKs is regulated both by cyclin binding and by phosphorylation of conserved residues. Reversible protein phosphorylation by protein kinases and phosphatases is a major regulatory mechanism of most cellular processes in eukaryotic organisms [2]. Progression through the G2/M phase transition in eukaryotes requires cyclin B/Cdk1 activity, which is regulated in turn through dynamic phosphorylation, a major regulatory mechanism of most cellular processes in eukaryotic organisms [3]. The phosphorylation status of threonine 14 (T14) and tyrosine 15 (Y15) of the catalytic subunit of CDKs regulates their activity and determines the timing of G2 and mitosis [4].

Phosphorylation by Wee1 on the Y15 residue in the ATP binding site blocks Cdk1 activity, whereas dephosphorylation by its antagonist CDC25 activates the enzyme, triggering the G2- to M-phase transition [4]. The opposite activities of Wee1 and CDC25 constitute the main switch for mitosis [5]. Wee1 was initially described in the fission yeast *Schizosaccharomyces pombe* as the target of mutations that allow cells to divide prematurely, thus producing cells half their usual length [6,7]. Later, one or more homologs have been found in all other eukaryotes examined so far, including *Saccharomyces cerevisiae* [8], humans [9], *Xenopus* [10], mice [11], *Drosophila* [12], and *Arabidopsis* [13]. Based on its sequence, it has been considered that Wee1 is an atypical tyrosine kinase included in the serine-threonine-specific family of protein kinases [7,14]. Wee1 contains three domains: an N-terminal regulatory domain, a central kinase domain, and a short C-terminal regulatory domain [9,12,15], and is regulated at multiple levels such as transcription [16], translation [17] and protein stability [18,19].

*Trypanosoma brucei*, causal agent of sleeping sickness in humans and nagana in cattle, is a protozoan parasite with a complex life cycle that involves different hosts and two forms of the parasite: bloodstream (in mammals) and procyclic (in insect vector) [20]. In both forms, the cell cycle has the usual sequential G1, S, G2 and M phases [21] but differs from that of other organisms by the presence of a kinetoplast cell cycle with an S phase (SK) and a phase of kinetoplast segregation preceding the nuclear S phase (SN) and mitosis [21,22]. Moreover, two other major organelles must be duplicated and segregated accurately before cell division: the basal body and the flagellum subtended from the basal body.

Molecular regulation of the *T. brucei* cell cycle has unique and unusual features. There is clear evidence that the trypanosomatid cell cycle is regulated by CDKs [23–26], although modulation of CDK activity may have evolved trypanosomatid-specific features. *T. brucei* possesses eleven cdc2-related kinases (CRK1-4 and CRK6-12). CDKs are activated by the binding of a cyclin partner and 10 cyclins (CYC2-11) are codified in the *T. brucei* genome [25,27,28]. 

Functional analysis of some of the cdc2-related kinases and cyclins of *T. brucei* has revealed their role in the regulation of the different cell cycle checkpoints. In both the bloodstream and procyclic forms, RNA interference (RNAi) of CRK1 or CYC2 enriched cultured in G1 phase cells [29–31]. The trypanosomatid G2/M phase transition is also regulated by the activity of mitotic CDK. The functional homolog of mammalian CDK1, CRK3, complexed with CYC6, is required for mitosis [25,30,32]. Although intracellular signaling events have not yet been described in detail for trypanosomatids, it is likely that tyrosine phosphorylation also plays a role in cellular processes as it does in higher eukaryotes. Phosphorylation on tyrosine residues is well documented in trypanosomatids [33–35]; although a key difference between host and parasite kinomes is the complete lack of protein kinases that map to the receptor tyrosine kinase and tyrosine kinase-like groups in these parasites. It has been proposed that tyrosine phosphorylation is likely due to the action of atypical tyrosine kinases such as Wee1 and dual-specificity kinases that can phosphorylate serine, threonine, and tyrosine [36]. Moreover, CRK3 contains a conserved tyrosine residue (Y34) in the same subdomain as the human CDK1 regulatory tyrosine (Y15) [23]. A large-scale phosphoproteomic analysis of the bloodstream [35] and procyclic forms of *T. brucei* [34] has shown that there is indeed phosphorylation on tyrosine residues on CRK3, CRK2 and CRK1, which could correspond to the conserved CDK1 canonical sequence motifs. As trypanosomatids transcribe most of their genes in large polycistronic units, signaling cascades in these parasites may function by post-transcriptional regulation. 

In the present study, we show that TbWee1 is a protein with sequence similarities to well-known Wee1-like kinases, expressed in both the procyclic and bloodstream forms of *T. brucei*. We demonstrate that the expression of TbWee1 in *S. pombe ∆Wee1* mutants is able to rescue the shortening of the cell cycle and promote the cell elongation, indicating that TbWee1 functionally complements the ΔWee1 phenotype in yeasts. Also, we showed that TbWee1 is an essential gene necessary for proper cell cycle progression and parasite growth in *T. brucei*.

## Materials and Methods

### *Trypanosoma brucei* cell culture

Cells of the procyclic form of *T. brucei* strain 29-13 [37] were cultured at 28°C in SDM-79 medium supplemented with 10% fetal bovine serum (FBS, PAA Laboratories GmbH), G-418 (15 µg/ml) and hygromycin B (50 µg/ml) in the culture medium to maintain the T7-RNA polymerase and tetracycline-repressor gene constructs in the cells.

### Expression of recombinant TbWee1 protein and antibody production

The human (NP003381.1) and *S. pombe* (NP 587933.1) Wee1 amino acid sequences were obtained from the Entrez Protein database using the NCBI web site (http://www.ncbi.nlm.nih.gov/sites/entrez?db=protein). Each sequence was used to search for homologous proteins in *T. brucei* in the TriTryp database (http://tritrypdb.org/tritrypdb/). Sequences were aligned using Multiple Sequence Alignment by CLUSTALW (http://align.genome.jp). The only Wee1 homolog coding region identified in the genome of the parasite was designated as TbWee1. It was amplified from genomic DNA obtained from *T. brucei* by PCR using the oligonucleotides 5´-GAATTCATGTTGGCGCCTAAAGGGG-3´ and 5´-CGGATATCCTAAAATTTTGCACTATCTCC-3´ (restriction sites corresponding to EcoRI and EcoRV are underlined) using *Pfu* polymerase (Stratagene, La Jolla, USA). *TbWee1* was cloned into the EcoRI and EcoRV restriction sites of the *Gateway* entry vector *pENTR* 2B (Invitrogen). The Gateway LR Clonase II Enzyme Mix kit (Invitrogen) was used to insert the *TbWee1* coding sequence into the destination vector pDEST17 (Invitrogen) to generate the recombinant protein with an N-terminal histidine tag. PCR-induced mutations were discarded by sequencing (Macrogen Inc., Seoul, Korea). *Escherichia coli* BL-21 (DE3) pLysS cells containing the full pDEST17-TbWee1 construct were grown in Luria-Bertani medium at 37°C to an OD at 600 nm (OD_600_) of 0.6. Expression of the *TbWee1* gene was induced by addition of 0.8 mM isopropyl-δ-thiogalactoside (IPTG), and the culture was further grown for 4 h at 37°C. The recombinant protein was obtained from inclusion bodies as follows: cells were harvested, resuspended in lysis buffer containing 50 mM Tris-HCl buffer, pH 8.0 and 20 mM NaCl and sonicated twice for 30 s (Heat Systems Ultrasonics, Inc.) followed by 1 min rest between cycles at 4°C between cycles. The cell extract was then subjected to seven freezing and thawing cycles and centrifuged at 14000x*g* for 30 min at 4°C. The pellet was resuspended in buffer containing 50 mM Tris-HCl buffer, pH 8.0, 20 mM NaCl and 8 M Urea, sonicated four times (4 pulses of 30 sec) followed by 1 min rest between cycles at 4°C and centrifuged at 14000x*g* for 30 min at 10°C. The supernatant obtained was loaded onto a nickel affinity chromatography (Ni–NTA agarose) (Qiagen, Germantown, MD, USA). Protein fractionation was performed according to the manufacturer’s instructions. The fractions containing the TbWee1 fusion protein were monitored by SDS-PAGE, pooled, dialyzed twice against 20 mM Tris-HCl, pH 7.8, 200 mM EDTA for 2 h, and once overnight in 20 mM Tris-HCl, pH 7.8. The purified recombinant protein was used as antigen to produce antibodies in mice.

### Generation and induction of RNAi cell lines

Primers for amplification of an RNAi target fragment were designed using the RNAit software tool (http://trypanofan.path.cam.ac.uk/software/RNAit.html). RNAi target fragments were PCR amplified using Phusion DNA polymerase (Finnzymes, Espoo, Finland). A 527-bp fragment of *TbWee1* was amplified from genomic DNA of the procyclic form of *T. brucei* by using the primers 5′- GGACTAGTCAAGGAGGTGAAGGAGCTT-3′ (SpeI site is underlined) and 5′-CCCTCGAGCACTGAACAAGTTCCCGGTT -3′ (XhoI site is underlined). PCR products were purified and cloned into the p2T7^Ti^-177 vector [38]. Cells of the 29-13 strain were transfected by electroporation as follows. Briefly, 10^8^ cells were harvested, washed twice with cytomix buffer (120 mM KCl; 0.15 mM CaCl_2_; 10 mM K_2_HPO_4_; 25 mM Hepes; 2 mM EGTA; 5 mM MgCl_2_; 2 mM ATP; 5 mM glutathione; pH adjusted to 7.6 with KOH) and suspended in 0.45 ml of cytomix buffer containing 10 μg of the NotI linearized constructs. Electroporation was carried out using a Bio-Rad electroporator with peak discharge at 1.6 kV and 25 μF of capacitance. The transfected cells were immediately transferred into 10 ml of SDM-79 supplemented with G418 and hygromycin. The transfectants were selected under 2.5 μg/ml phleomycin with individual cells cloned by limiting dilutions, generating independent Wee1 RNAi cell lines. The stable transfectants thus obtained were then induced with 2.5 µg/ml tetracycline to switch on the T7 promoter, to initiate theTbWee1 RNAi. Cells in the presence (+Tet) or absence (−Tet) of tetracycline were counted daily and cumulative growth curves for each clone were plotted on a logarithmic scale.

### Northern Blot Analysis

Total RNA was obtained from *T. brucei* using TRIzol reagent (Invitrogen, Carlsbad, California, USA) according to the manufacturer's instructions and Northern blotted as previously described [39]. Probes for TbWee1 were full-length open reading frames (ORF). The probes were radiolabeled with [α-^32^P] dCTP (10^9^ cpm pmol^−1^, NEN) using the Prime-a-Gene Labeling System (Promega, Madison, WI, USA). Quantification was performed using a Storm 820 Phosphorimager (Amersham Pharmacia Biotech, Sweden) and ImageQuant software. Ribosomal RNA was used to measure loading. 

### Flow cytometry analysis of RNAi cell lines

Cell samples for flow cytometry analysis were prepared as follows. Briefly, 10^6^ cells harvested at different times by centrifugation at 600 g for 10 min were washed twice with cold PBS plus 2mM EDTA. The cell pellets were resuspended in 200 µl PBS plus 2mM EDTA and fixed by adding 1.5 ml of 70% ethanol in PBS dropwise while vortexing. Samples were stored at 4°C overnight. The fixed cells were washed with PBS and then suspended in 1 ml of PBS containing RNase A (10 mg/ml) and propidium iodide (PI) (20 mg/ml). The mixture was incubated at 37°C for 30 min and then analyzed using a FACSCalibur flow cytometer (BD Biosciences, Franklin Lakes, New Jersey, USA). Percentages of cells at different phases of the cell cycle were evaluated by ModFitLT software (Becton Dickinson). 

### Analysis of the configurations of nuclei and kinetoplasts

*T. brucei* cells were harvested, washed three times with PBS, and fixed on slides with cold methanol at −20°C for 20 min. Slides were washed with PBS in the presence of 1 μg/ml of DAPI. Subsequently, cells were examined with an Olympus phase-contrast and fluorescence microscope to tabulate the numbers of nuclei and kinetoplasts in individual cells in populations of more than 200 cells in each sample.

### Hydroxyurea-induced synchronization of *T. brucei* cell cycle

Synchronization of the procyclic forms of *T. brucei* in S phase using hydroxyurea (HU) was achieved essentially as described in Chowdhury et al. [40]. Briefly, 10 ml culture (2.5 x 10^6^ cells/ ml) was incubated in medium containing 0.2 mM HU for 12 hours. Then the HU was removed by centrifugation (1200 g, 10 min) and the cells were washed twice with medium at room temperature. After washout of HU, cells continued to be cultured for 12 hours. To assess synchrony, we fixed cells every 2 h, stained them with propidium iodide, and conducted flow cytometry as described in section 2.6. TbWee1 protein was detected in the different cell cycle stages by Western blotting using the anti-TbWee1 antiserum (1:1000). 

### Overexpression of TbWee1 in *Schizosaccharomyces pombe*

The *TbWee1* gene was expressed in *S. pombe* strain FY7283 (*∆Wee1*: *h* ^-^ *, wee1::ura4* ^*+*^ *, leu1-32, ura4-D18*, kindly provided by YGRC) (http://yeast.lab.nig.ac.jp/nig). The coding sequence of *TbWee1* was amplified using primers 5´-CCGCTCGAGATGTTGGCGCCTAAAGGGG-3´ and 5´-CGCGTCGACCTAAAATTTTGCACTATC -3´. The PCR product was ligated into the XhoI and SalI sites of pREP3X [41] and expressed under the control of the strong promoter *nmt1*, repressible by thiamine [42]. *S. pombe* competent cells were electroporated with pREP3X-SpWee1 (Wee1 from *S. pombe*), pREP3X-TbWee1 (Wee1 from *T. brucei*) or pREP3X alone. Transformants were selected on minimal medium [43] supplemented with 70 mg/L uracil and 70 mg/L adenine, plus 10 µM thiamine. Expression was induced for 4 days at 28°C by growing the yeast cells in minimal medium in the absence of thiamine, or repressed by the addition of 10 µM thiamine. Images were taken using an Olympus BX41 light and fluorescence microscope with an Olympus DP71 digital camera and capture software (Olympus America, Inc., Center Valley, PA, USA). Cell lengths were measured using ImageJ software (http://rsbweb.nih.gov/ij/). Experiments were repeated using several independent transformants, all yielding similar results. For DAPI staining, 1 ml of exponentially growing *S. pombe* cells were centrifuged and fixed in 100 % methanol and incubated for 1 hour at -20°C. The cells were concentrated to 30 µl of methanol, a drop of 5 µl was dried in a glass cover for 5 min at room temperature and incubated with 0.5 µg/ml DAPI (Molecular Probes).

### Phosphorylation assays of recombinant protein TbWee1 expressed in baculovirus system

Full-length TbWee1 fused to an N-terminal hexahistidine-tag expressed in baculovirus where generated using the Bac-to-Bac® Expression System (Invitrogen). The TbWee1 coding region was amplified as described in section 2.1 and the transfer plasmid was generated using pFastBac^TM^ (Invitrogen). *Spodoptera frugiperda* Sf-9 insect cells were propagated in Grace’s medium (Gibco) at 27°C and infected at a multiplicity of infection (MOI) of 1. The recombinant protein was purified using nickel affinity chromatography (Ni–NTA agarose) (Qiagen) according to the manufacturer´s procedures. The recombinant protein rTbWee1 thus obtained was detected both with the TbWee1 and polyhistidine antibodies, and used for phosphorylation assays. These assays were performed with 40 µg of rTbwee1 either with 0.2 mg/ml poly-(glu:tyr) (Sigma-Aldrich, St. Louis, Missouri, USA), a general substrate for tyrosine protein kinase, and analyzed with P81 phosphocellulose papers [44], or with 0.5 mg ml^-1^ of histone H1 as previously reported [39]. The last reactions were stopped with 5x Laemmli´s buffer, analyzed on 12% SDS-polyacrylamide gels and transferred to an Amersham Hybond-ECL nitrocellulose membrane (GE Healthcare, Little Chalfont, Buckinghamshire, UK), according to the manufacturer’s instructions. Membranes were exposed to X-Omat Kodak films (Sigma-Aldrich) or the signal was scanned with a Storm 820 Phosphorimager (Amersham) [39]. Alternatively, the blotted membranes were analyzed with anti-phosphotyrosine (1:1000) (Transduction Laboratories, Lexington, Kentucky, USA) and with anti-polyhistidine (1:5000) (Sigma-Aldrich) antibodies. rTbWee1 autophosphorylation was determined according to the method of Ferrell and Martin [45], as described previously [44] (data not shown). 

## Results

### Identification of Wee1 homolog in trypanosome genomic database

A systematic search for a potential *Wee1* gene homolog in *T. brucei* was performed using the human and fission yeast *Wee1* sequences described. A single homologous gene for this kinase was found in *T. brucei* chromosome 4. The ORF corresponding to this putative *T. brucei* Wee1 kinase-like homolog was designated as *TbWee1* and recorded under the GenBank accession no. JN083854. The identification of only one sequence by BLAST indicates that *TbWee1* is a single-copy gene. This predicted TbWee1 kinase-like homolog is encoded by an 1812-bp ORF and consists of 603 amino acids with a predicted molecular mass of 66.22 kDa and a calculated isoelectric point of 6.57. 

**Figure 1:**
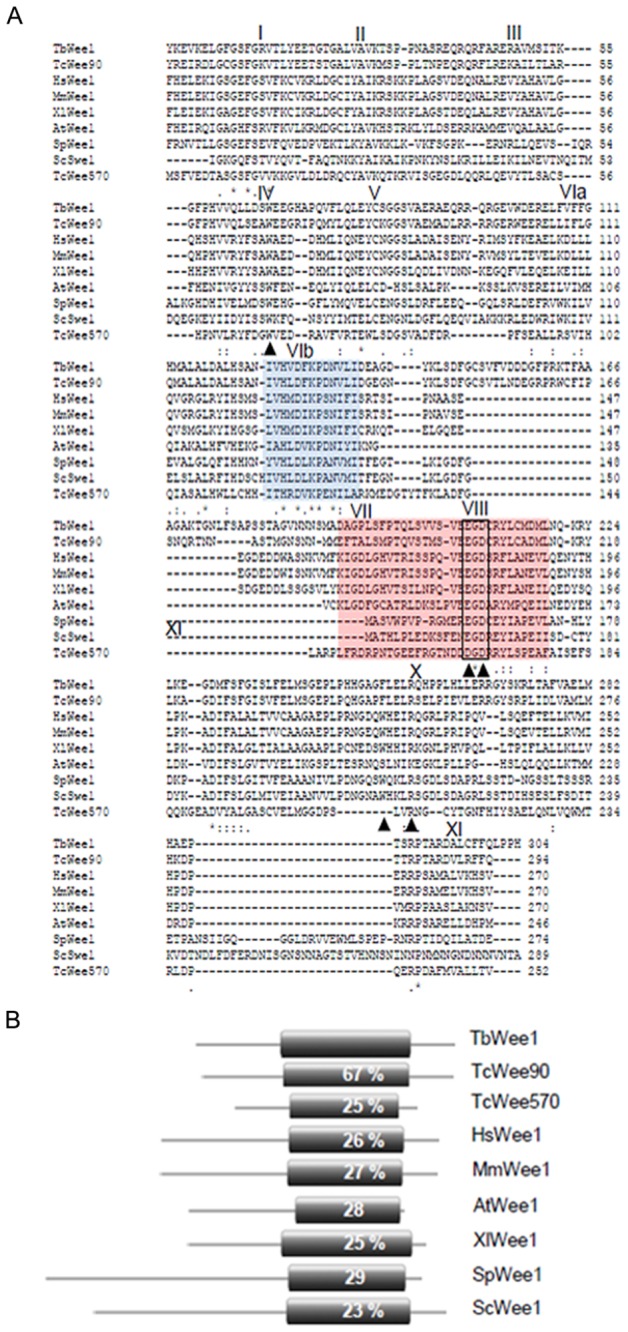
Sequence comparison between TbWee1 and homolog proteins in other species. (A) Multiple sequence alignment of the catalytic domains of the putative *Trypanosoma brucei* Wee1 protein with other Wee1-like kinases. Amino acid sequences were aligned using the ClustalW2 program (http://www.ebi.ac.uk/clustalw). Identities are indicated by asterisks below the sequence. Conserved substitutions are marked with two vertical dots and semi-conserved substitutions are marked with a single dot. Dashes represent gaps introduced for optimal alignment. The 11 conserved subdomains are designated by Roman numerals [45,46]. The catalytic and activation segments are indicated with a blue and pink box respectively. A black box indicates the conserved EGD motif. Triangles indicate amino acids that are conserved in all known members of the Wee1 kinase family, but not in other eukaryotic protein kinases. Sequences shown are for Wee1A kinase of *Trypanosoma* *brucei* (TbWee1, JN083854), humans (HsWee1, NP003381.1), mice (MmWee1, NP033542.2), *Schizosaccharomyces* *pombe* (SpWee1, NP587933.1), *Saccharomyces* *cerevisiae* (ScSwe1, NP012348.1), *Arabidopsis* *thaliana* (AtWee1, NP171796.1), *Xenopus laevis* (XlWee1, NP001081784.1), and *Trypanosoma cruzi* (TcWee90, JN573306; TcWee570, JN257712). (B) Comparison of TbWee1 with other protein kinases. The position of the putative protein kinase domain is shown in black and numbers represent the percent amino acid identity with this region of the predicted TbWee1.

An alignment of the catalytic domain of TbWee1 with orthologue sequence from several organisms is shown in Figure 1A. A search in the *Trypanosoma cruzi* genome database revealed that two putative Wee1 homologs are present in this trypanosomatid. These genes were termed *TcWee90* (GenBank accession no. JN573306) and *TcWee570* (GenBank accession no. JN257712) and share 67 % to 25 % sequence identity with TbWee1 respectively (Figure 1B). The low level of sequence homology between TbWee1 and TcWee570 suggests that they may have originated from different subfamilies. The percentages of identity between TbWee1 and other Wee1 kinases range from 23 to 29% (Figure 1B). All stated amino acid identities are based on BlastP scores. Apart from the catalytic region, there was little homology between TbWee1 and other protein kinases. This is consistent with the fact that there are no obvious sequence homologies between N-terminal domains of Wee1-like proteins isolated from divergent species.

Eleven subdomains of the kinase catalytic domain with a high degree of conservation have been described [46,47] and all of them are present in TbWee1 (Figure 1A). The TbWee1 catalytic domain (residues 189-483) has the typical glycine-rich loop (GxGxxG, residues 207-212) and Lys229, which are involved in ATP binding. The catalytic segment (residues 324-336), which is highly conserved, contains the active site motif HxD and closely matches the Wee1 protein kinase consensus sequence IVHxDLKPxNIx. This motif contains the invariant residues Asp328 and Asn333, involved in the phosphotransfer reaction. Asp328 is presumably the catalytic base that accepts the proton from the attacking hydroxyl group of the substrate. The amino acid Lys330 possibly neutralizes the negative charge of the γ-phosphate and thereby facilitates the phosphotransfer [47]. The activation loop (residues 390-418) is 29 residues long and contains the Glu407-Gly408-Asp409 triplet motif (EGD motif), which is found exclusively in subdomain VIII of the Wee1 protein kinase family [8]. Another diagnostic feature found in all members of the Wee1 kinase family is the conserved residues Trp in subdomain IV and a Trp and Arg in subdomain X [10]. These residues are present in TbWee1 at conserved positions, although Trp in subdomain X is substituted by the hydrophobic amino acid Phe (Figure 1A). No N-terminal signal peptide, transmembrane domains or localization motifs were identified in the TbWee1 sequence. The phylogenetic relationship between the sequences used in the multiple sequence alignment is shown in Figure 1C. Phylogenetic analyses were conducted using MEGA version 5 [48]. This phylogeny, based on the kinase domains of Wee1, shows three main groups: animal and plant Wee1, fungal Wee1 and trypanosomatid Wee1, supporting the existence of a novel trypanosomatid Wee1 family with unique features.

All together, these characteristics support that TbWee1 is a putative *Wee1* kinase in *T. brucei*.

### Down-regulation of TbWee1 expression inhibits the cell cycle of the procyclic form of *T. brucei*

**Figure 2:**
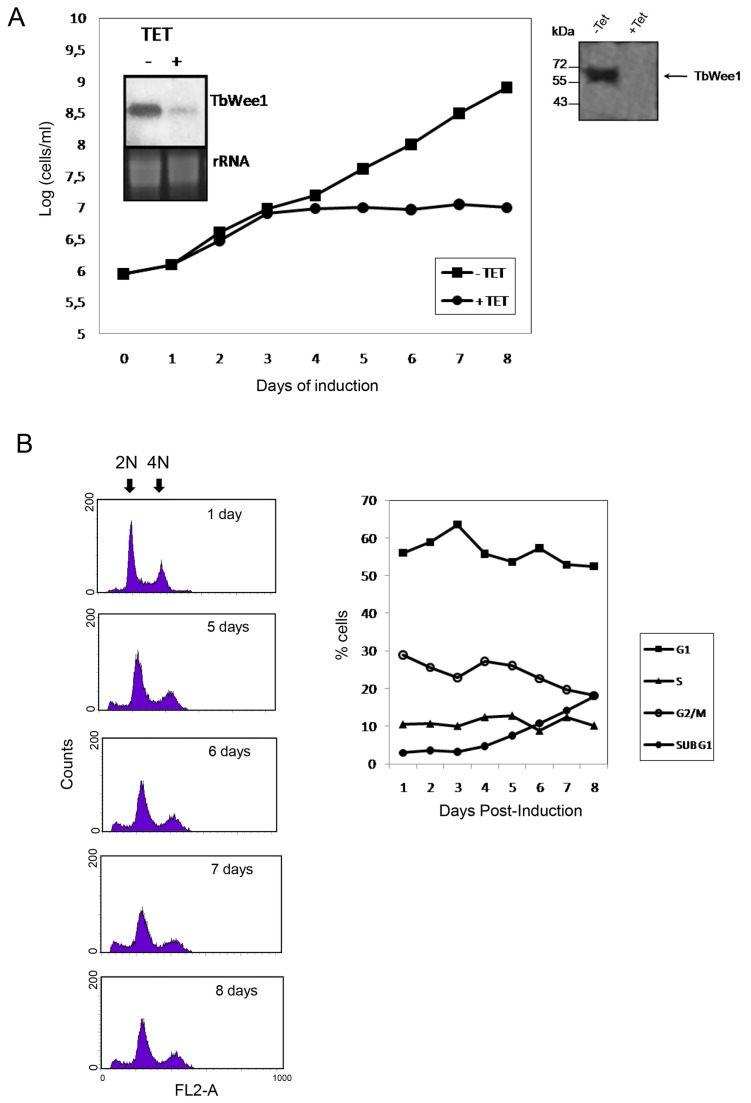
Effects of TbWee1 knockdown on the procyclic form of *T. brucei* cells. (A) Cells of strain 29-13 harboring the TbWee1-RNAi construct were incubated in culture medium with (+ Tet) or without (-Tet) 2.5 µg/ml tetracycline at 28°C. The cell growth rate was monitored daily, and the cell number was plotted in a logarithmic scale. The insets show the intracellular mRNA level after 3 days of RNAi as monitored by Northern blot. RNAr was used as loading control. Western blot of extracts of induced and non-induced cells were analyzed with anti-TbWee1 antibody (Right inset). (B) Time course of RNAi-induced *T. brucei* procyclic-form. Cells were stained with propidium iodide and subjected to FACS analysis to measure DNA content. The percentages of cells in G1, S and G2/M phases were determined with the ModFitLT software and plotted on the right panel.

To investigate whether TbWee1 depletion affected cell proliferation, a stable tetracycline inducible RNAi cell line was generated using a portion of *TbWee1* that has no significant sequence identity with other genomic sequences of *T. brucei*, clones into the p2T7^Ti^-177 vector. The effect of RNAi on Wee1 gene expression was examined by Northern blot. The level of Wee1 mRNA was reduced by 80 % after 3 days of RNAi induction (Figure 2A, inset). The expression of TbWee1 was explored by Western blot, and our results indicate that the protein was reduced in the induced cells (+Tet) (Figure 2A, right panel).

To investigate the effect on cell growth of TbWee1, TbWee1-deficient parasites (as control) were counted daily during an 8-day incubation period (Figure 2A). Non-induced cultures were used as control. Since each independent clone gave essentially the same phenotype, only the data for one clone are shown. Cell growth was clearly inhibited after 4 days of induction, and 8 days post-transfection cell viability was reduced 4-fold upon silencing of Wee1 compared to non-induced RNAi cell lines. The viability of TbWee1-depleted cells was inhibited by more than 77 % upon silencing of Wee1. The significant inhibition of cell growth suggested that TbWee1 could play an essential role in the procyclic form of *T. brucei.*

### Depletion of TbWee1 modifies the cell cycle progression of the procyclic form of *T. brucei*

To assess the effect of Wee1 loss of function on the cell cycle at different times, the percentages of cells at different phases of the cell cycle were analyzed by flow cytometry (FACS). When the expression of Wee1 was knocked down for 8 days, FACS analysis of the DNA content of a *T. brucei* culture showed a decrease of 10% in G2/M-phase cells. This was accompanied with only slight changes in the G1-phase population, whereas the S-phase cells remained relatively unchanged (Figure 2B). The emergence of a sub-G1 peak (from ~2 to 18 %) was evident in tetracycline-induced cells when compared to non-induced ones. 

**Figure 3:**
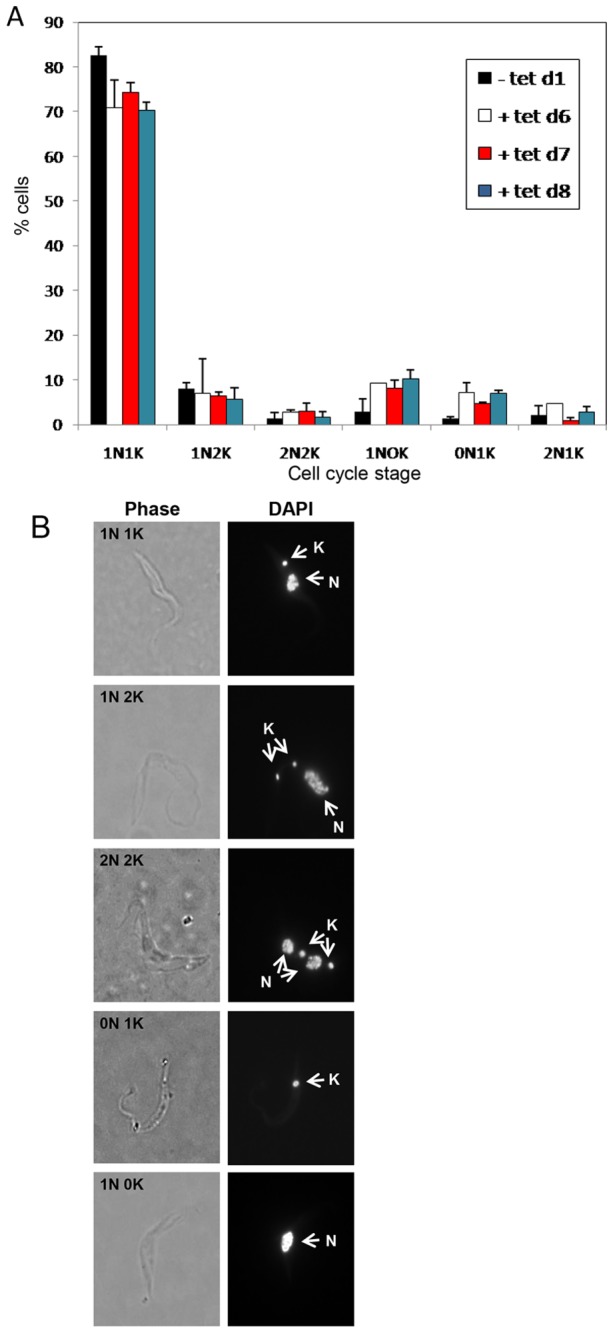
Morphological phenotypes of Wee1-deficient *T. brucei* procyclic-form cells. Samples of the TbWee1-depleted cells taken at different times were stained with DAPI and examined by fluorescence microscopy. (A) Analysis of the numbers of nuclei and kinetoplasts as determined by DAPI staining. Data are presented as the mean percentages ±S.E. of the total population counted (> 200 cells in each of three independent experiments). (B) Wee1-deficient cells viewed by phase-contrast and fluorescence microscopy. N: nucleus, K: kinetoplast.

A time course of the effect on cell division and kinetoplast segregation of TbWee1 depletion was monitored by using DAPI staining to visualize nuclei and kinetoplasts (Figure 3A). The cells in G1 and S phases seemed to have one nucleus and one kinetoplast (1N1K). Since K division precedes nuclear division, cells in the G2/M phase have one nucleus and two kinetoplasts (1N2K), and cells undergoing cytokinesis have two nuclei and two kinetoplasts (2N2K). In the control non-induced Wee1 culture, 82% of the cells had a normal-size single nucleus and a single kinetoplast (1N1K), while the rest of the cells (18%) were mainly either 1N2K or 2N2K (Figure 3 A, B). After 6 days of RNAi induction, the 1N1K population decreased significantly from 82 to 70%, whereas the 1N2K and 2N2K populations showed no apparent changes. However, the average size of every individual nucleus in 1N2K cells appeared significantly enlarged. These cells were named1N*2K to be distinguished from the regular 1N2K (Figure 3B). The population of enucleated cells (zoid), each containing a single kinetoplast (0N1K), increased from 0% to 7% of the population, while 1N0K cells increased from about 3 to 10% (Figure 3A, B). The appearance of 1N*2K dividing cells showing the formation of abnormal 1N1K and 0N1K cells further confirms the mitosis defect in Wee1-depleted cells.

RNAi of TbWee1 resulted in an increase in the proportion of cells with a <1C DNA content (subG1 peak). This agrees with the observed karyotype of the cells, showing an increase in the number of zoids and cells with 1N0K. These could be the daughter cells derived from the division of 1N*2K cells to give rise to 1N1K cells and zoids. This observation suggests that cytokinesis can be driven by kinetoplast segregation alone without mitosis. 

### TbWee1 can rescue *S. pombe* ΔWee1 mutants

The Wee1 gene was originally identified as a genetic element that controlled the size at which *S. pombe* cells entered mitosis [6]. Loss of Wee1 activity causes cells to enter mitosis before sufficient growth has occurred. Therefore, cytokinesis produces two daughter cells of abnormally shortened length (ΔWee1 phenotype). Conversely, increasing the gene dosage of Wee1 causes delayed entry into mitosis and an increase in cell length. This indicated that the levels of Wee1 activity determine the timing of entry into mitosis and have strong effects on cell size [7].

In order to investigate if TbWee1 possibly belongs to the Wee1 kinase family, we next attempted to rescue the growth defect of a *S. pombe* strain lacking *wee1* (*∆Wee1*). *TbWee1* gene was expressed in the fission yeast under the control of the thiamine-repressible *nmt1* promoter using was vector pREP3X [49].

**Figure 4:**
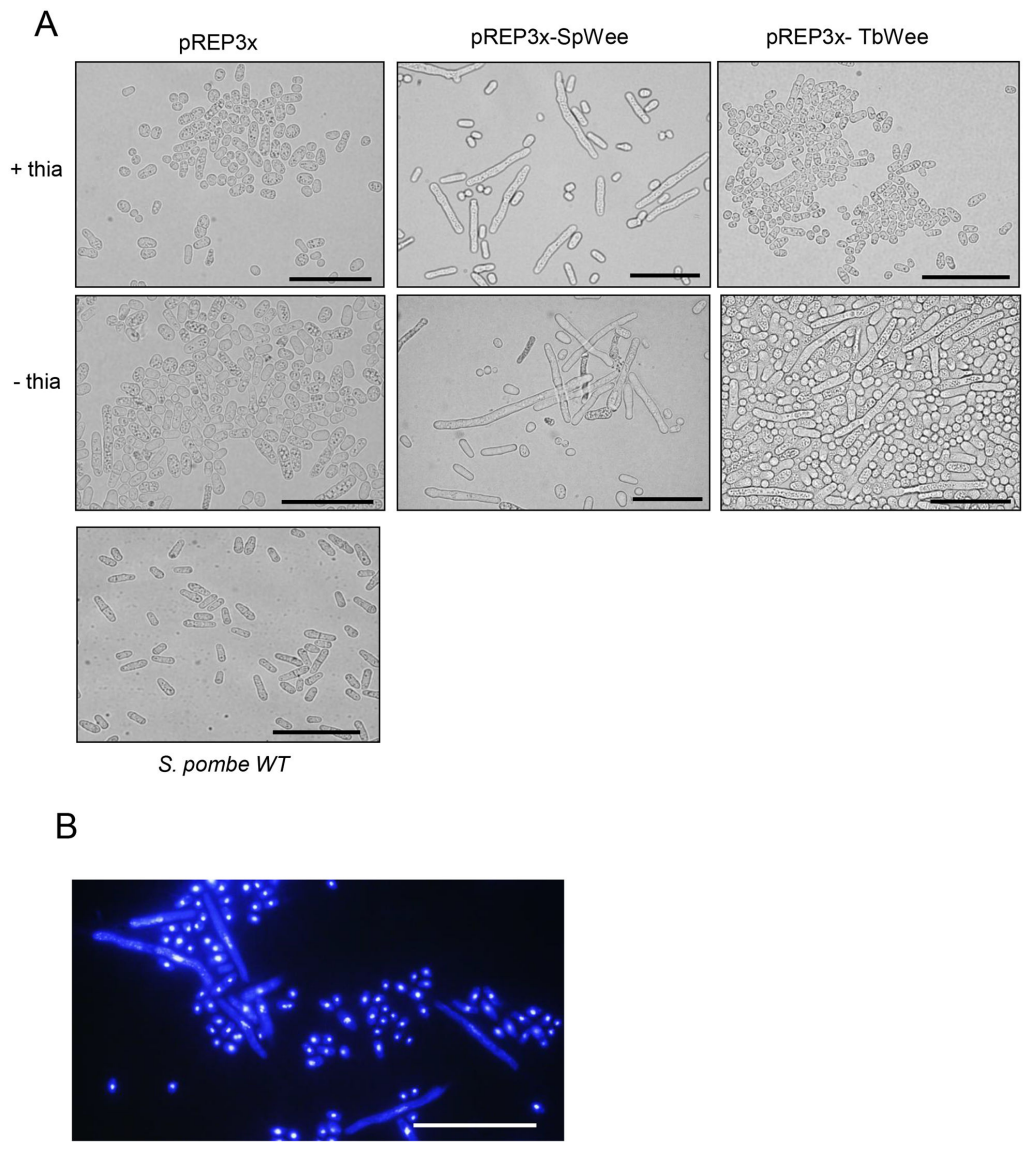
Rescue of a *Schyzosaccharomyces pombe* Wee1 mutant by TbWee1. (A) *S. pombe* ∆Wee1 mutants were transformed with the pREP3x vector or with pREP3x in which *S. pombe* Wee1 or TbWee1 was cloned. Fission yeast were cultured on solid media in the presence (+thia) or absence (-thia) of 15 µM thiamine. Lower panel: *S. pombe* cells expressing wild type Wee1. (B) DAPI staining of *S. pombe* cells transformed with pREP3x TbWee1 grown in the absence of thiamine. Average cell size in *S. pombe* yeasts complemented with TbWee1 was 16,3 µm, with 55% of the population in the 10-15 µm range. Average cell size in control *S. pombe* yeasts was 10,4 µm, with 57% of the population in the 5-10 µm range. n=100. Bar= 100 µm.

*∆Wee1* mutants expressing TbWee1 exhibited cell cycle arrest manifested by an increase in cell length after washing out thiamine repression from the cell culture. This same phenotype was observed when Wee1 from *S. pombe* was overexpressed in the same cells (Figure 4A). This long-cell phenotype was not seen when expression of either Spwee1 or TbWee1 was repressed with thiamine or when ΔWee1 cells were transformed with and empty vector (Figure 4A). Moreover, over-expression of TbWee1 caused *S. pombe* cells to elongate and reach a size even larger than that of wild-type cells, indicating that the effect of TbWee1 on the growth of ∆Wee1 cells is dose-dependent (Figure 4A).

To verify that the long cell phenotype induced by TbWee1 overexpression was not due to pseudohyphal growth (a phenotype characterized by the lack of separation of daughter cells after mitosis and septation [50]), the position and the number of nuclei per *S. pombe* cell were examined by DAPI staining. Only one nucleus per cell and no septum were seen in ΔWee1 cells expressing TbWee1 (Figure 4B), indicating that an increased cell length was not due to pseudohyphal growth and confirming the ability of TbWee1 to rescue the mutant *∆Wee1* phenotype. 

These data demonstrate that TbWee1 exhibits functional properties that are characteristic of Wee1 kinases and can fulfill a role similar to that of the fission yeast Wee1. This supports the notion that TbWee1 could be the trypanosomal functional homolog of Wee1 kinase.

### Autophosphorylation activity of TbWee1

**Figure 5:**
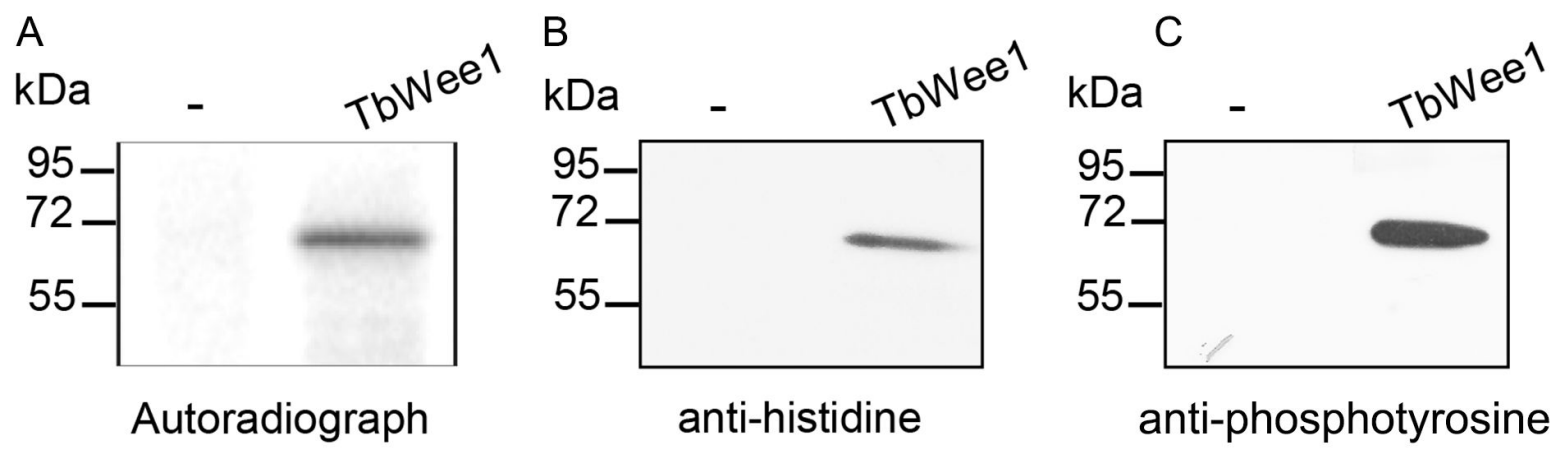
Autophosphorylation of recombinant TbWee1-6xHis fusion protein. Phosphorylation reactions were performed in the absence or presence of 40 µg of rTbWee1-6xHis expressed in the baculovirus system, in a mixture containing 5 µCi [γ-32P]-ATP (6000 Ci/mmol, NEN) for 10 min at 30°C. Reaction products were resolved by 12 % SDS-PAGE and visualized by autoradiography. Membranes were revealed with anti-histidine tag antibody (1:5000) and anti-phosphotyrosine antibodies (1:1000).

Many protein kinases are known to undergo autophosphorylation. To test whether this was the case for TbWee1, purified recombinant TbWee1-6xHis fusion protein obtained from the baculovirus system was incubated with [γ-^32^P] ATP in the presence of Mg^2+^ and Mn^2+^. Analysis by SDS-PAGE/autoradiography revealed the presence of a labeled band with the same molecular mass (~ 70 kDa) as the fusion protein (Figure 5A). Although the purified recombinant TbWee1-6xHis fusion protein was able to autophosphorylate *in vitro*, there was no detectable kinase activity when non-specific substrates such as histones (H1 and a mixture of histone HII) or the synthetic peptide poly-(glu-tyr) (Sigma-Aldrich), a general substrate for tyrosine kinase, were present in the reaction mixture. Western blot using the anti-histidine antibody confirmed that the labeled band was TbWee1 (Figure 5B). In addition, the autophosphorylated TbWee1 was recognized by an anti-phosphotyrosine antibody, indicating that some or all the phosphorylations occurred on tyrosine residues (Figure 5C). 

### Detection of the native TbWee1 protein in parasite extracts

**Figure 6:**
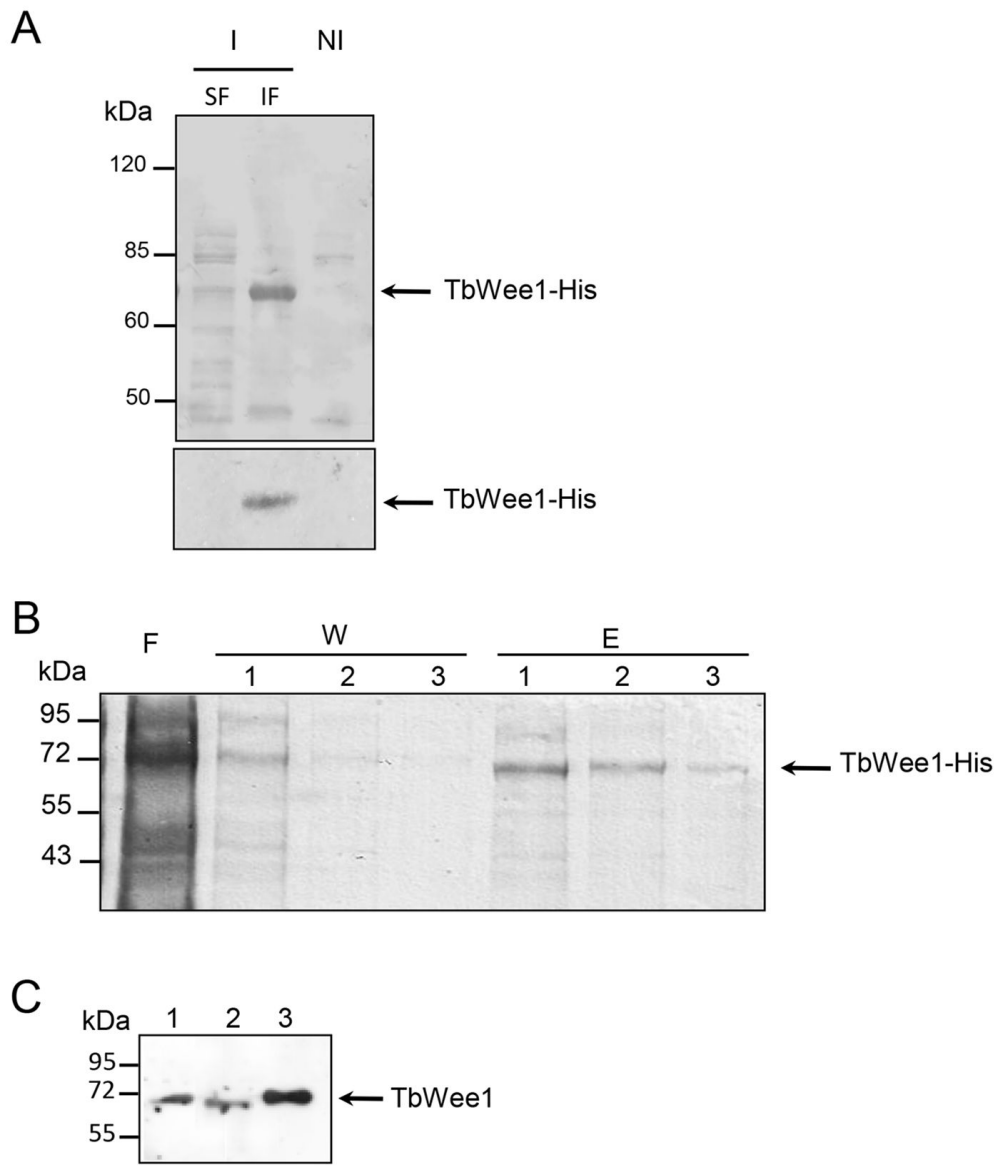
Amplification and purification of TbWee1-6xHis fusion protein to produce anti-TbWee1 polyclonal antibodies. (A) SDS–PAGE analysis of lysates obtained from *Escherichia coli* IPTG.induced (I) and non-induced (NI) cell cultures transformed with pDEST17-TbWee1. Upper panel: Coomassie Brilliant Blue staining. Lower panel: Western-blot analysis with anti-histidine tag antibody (1:5000). The arrow indicates the position of the ~70 kDa recombinant protein. (B) Purification steps of rTbwee1-6xHis fusion protein were monitored by 12 % SDS-PAGE analysis. The lysate was loaded onto a Ni-agarose column. Flow-through (F), washes (W, 1–3), and eluted fractions (250 mM imidazol, E 1–3) were analyzed by 12% SDS-PAGE stained with Coomassie Brilliant Blue. (C) Western blot analysis of rTbWee1 expression with polyclonal anti-TbWee1 antibody. Protein extracts of the *T. brucei* bloodstream form (lane 1), the *T. brucei* procyclic form (lane 2) and the recombinant TbWee1-6xHis (lane 3) were separated by 10 % SDS-PAGE and electroblotted on nitrocellulose membrane. Blots were incubated with anti-TbWee1 polyclonal antibody (1:1000) and revealed by chemiluminescence.

The expression pattern of TbWee1 protein was studied in the procyclic and bloodstream life cycle stages of the parasite, using antibodies obtained against the recombinant TbWee1 protein. In protein extracts from IPTG-induced *E. coli* cultures (Figure 6A, upper and lower panels), a strong ~70 kDa band was detected by Coomassie blue staining and Western blot analysis with the anti-histidine antibody. The recombinant fusion protein was only detected in the insoluble fraction (IF), and so the enzymatic activity of TbWee1 could not be determined. The purified TbWee1-6xHis fusion protein (E2, Figure 6B) was used to produce anti-TbWee1 polyclonal antibodies in mice.

The anti-TbWee1 antiserum recognized the ~70-kDa protein in total cell extracts of the procyclic and bloodstream forms (Figure 6C), supporting that Wee1 protein is present in both life cycle stages of the parasite.

### TbWee1 protein is only detected during the G2/M stage of the cell cycle

**Figure 7:**
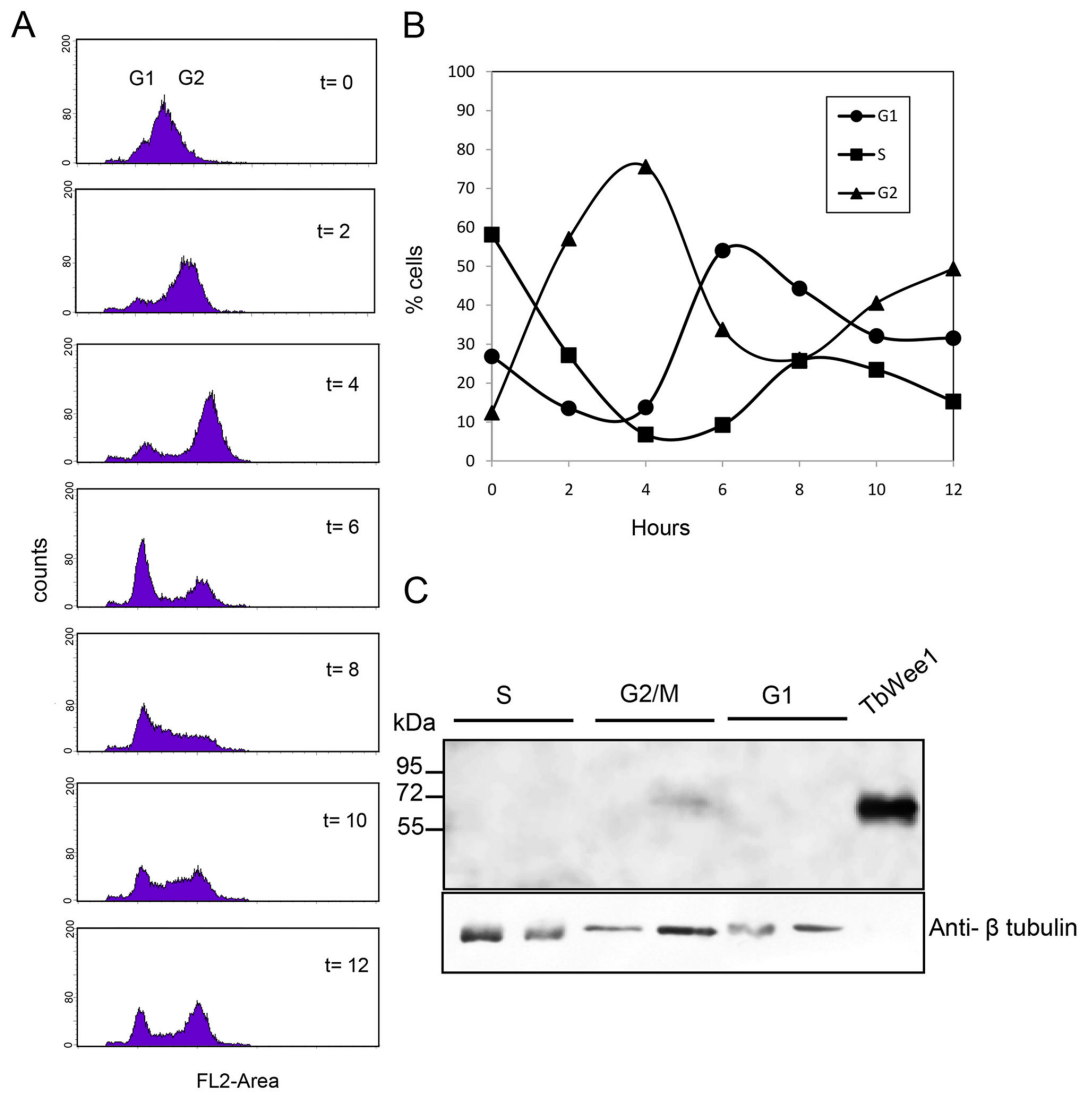
Synchronization of procyclic *T. brucei* and analysis of TbWee1 protein expression during different stages of the cell cycle. (A) Cells were synchronized with 0.2 mM hydroxyurea for 12 h, then HU was washed out and the cells were stained with propidium iodide and analyzed for 12 hs by flow cytometry performed every 2 h. (B) The percentages of cells in G1, S and G2/M phases were determined by the ModFitLT software. (C) Protein extracts from S, G2/M and G1 were separated by 12 % SDS-PAGE and electroblotted on nitrocellulose membranes. His-tagged recombinant TbWee1 (10 µg) was used as a positive control. Blots were incubated with anti-TbWee1 polyclonal antibody (1:1000) (lanes 1, 3, 5: 20 µg; lanes 2, 4, 6: 40 µg). An antibody recognizing β-tubulin (1:5000) was used as the load control.

To investigate whether TbWee1 protein levels fluctuated during the cell cycle, the expression pattern of TbWee1 protein was studied in all cell cycle stages of the parasite. *T. brucei* procyclic forms were synchronized with HU according to the new method of Chowdhury et al [40]. Synchrony was achieved through HU-induced depletion of the dNTP pool, resulting in a transient accumulation of cells at the G1 to S transition. After removal of HU, the cells underwent a short lag period and then progressed synchronously through the cell cycle. At different times after the release from the cell cycle blockage, cells were harvested and analyzed by FACS and immunoblot. Analysis of the DNA content indicated that cells progressed synchronously through different phases of the cell cycle (Figure 7A, B). Immunoblot analysis with anti-TbWee1 polyclonal antibody showed that the TbWee1 signal was present in low amounts in G2/M stage (Figure 7C) and could not be detected in G1 or S phases. A total of 40 µg protein extract was necessary to detect TbWee1 at very low levels in G2/M stage (Figure 7C) so TbWee1 protein cycles but even at G2/M phase its abundance is low.

## Discussion

Although considerable progress has been made on identifying molecular regulators of the cell cycle of *T. brucei*, many regulators undoubtly remain to be identified. Orthologs of many conserved protein kinases, such as CDKs, mitogen-activated protein kinases (MAPKs), Aurora and Polo-like kinases are present in *T. brucei*, although their functions are often divergent [51]. Although phosphorylation of protein tyrosine residues regulates important cell functions in higher eukaryotes, the roles of this post-translational modification is largely unknown for *T. brucei* [34,35]. No receptor-linked tyrosine kinases have been found in the trypanosomatid kinome, and tyrosine phosphorylation is likely carried out by dual-specificity protein kinases [34,36]. It has been reported that homologs of Wee1 kinase are present in the trypanosomatids but no homologs of Myt1 (a related member of the Wee family kinases), CDC25, Tome1 (an SCF type-E3 ligase that targets Wee1 for degradation at the onset of mitosis) and CDK inhibitor proteins have been found [36]. This suggests that other mechanisms have evolved to regulate the activity of CRKs in trypanosomatids. 

The goal of this study was to identify a homolog of Wee1 kinase in *T. brucei*. Focusing on several key issues relevant to the structure and function of Wee1 family, a search on the TriTryp database revealed a homolog that we named TbWee1, with a kinase domain similar to that of Wee1 homologs from other eukaryotes. TbWee1 coding sequence bears conserved residues shared by all kinases, as well as the signature motif “EGD” previously noted for the Wee1 kinase family [8,52]. Although Wee1 was functionally characterized as a tyrosine kinase, its primary amino acid sequence most closely resembles serine/threonine kinases such as Chk1 and cAMP-dependent kinases in structure and primary amino acid sequence [14].

In pair-wise comparisons, TbWee1 is more closely related to trypanosome Wee1 than to animal, yeast or plant sequences. All of these Wee1-like kinases share a similar structure with a C-terminal kinase domain and a less conserved, N-terminal domain. The protein sequences of the large N-terminal domains of Wee1 homologs from *S. pombe*, the budding yeast *S. cerevisiae*, the fruit fly *Drosophila melanogaster*, *Xenopus*, and humans are highly divergent [7–10,16]. These non-catalytic regions of the protein are thought to play important roles in the regulation, protein-protein interaction, and subcellular localization of Wee1 kinases [53–57].

In this report, we show that TbWee1 exhibits functional properties that are characteristic of Wee1 kinases. The Wee1 gene was originally identified as a genetic element that controls the timing of entry into mitosis and the size at which *S. Pombe* enters this cell cycle stage [6]. Loss of Wee1 activity causes cells to enter mitosis before sufficient growth has occurred, producing two daughter cells of abnormally small size (Wee1 phenotype). Conversely, increasing the gene dosage of Wee1 causes delay entry into mitosis and an increase in cell size [7]. We were able to complement a Wee1 mutant of *S. pombe*: yeasts expressing TbWee1 showed an increase in length compared to empty vector controls. Notably, these long-cell phenotypes were also obtained when human, *Drosophila* and maize homologs were over-expressed in *S. pombe*, and are indicative of G2 arrest [9,58]. Although the *Arabidopsis* and tomato (*Solanum lycopersicum*) Wee1 gene are unable to complement mutations in their yeast homolog, their overexpression inhibits cell division in fission yeast [13,59]. Although results from complementation experiments in yeast do not constitute unequivocal proof that TbWee1 has the same functions in *T. brucei* as Wee1 in yeast, they represent the first indication for a cellular function for a Wee1 protein kinase in trypanosomes.

Second, we investigated whether TbWee1 activity behaved as related Wee1 kinases from other eukaryotes, using a small amount of the rTbWee1 protein obtained from baculovirus. Autophosphorylation has been shown to regulate many protein kinases [60]. All Wee1 homologs have tyrosine residues in the amino-terminal region and most Wee1 homologs have been reported to undergo autophosphorylation [7–10,12,16,61–64]. TbWee1-His protein was able to autophosphorylate, as the same band that is recognized with anti-His tag and anti-phosphotyrosine antibodies resulted labelled upon incubation with [γ-^32^P] ATP. This modification could change the protein kinase activity in the entire cell. However, the protein kinase activity of this protein was not detectable. This could be due to the fact that this family of protein kinases has very specific substrate requirements [65].

Functional analysis of the cell cycle indicates that Wee1 is a key player that serves as a mitotic inhibitor in the intricate network of kinases and phosphatases that regulate G2 progression [16,66]. Antibodies raised against TbWee1 showed that this protein kinase is present in the proliferative procyclic and bloodstream slender forms of parasites. Using synchronized TbWee1 cells, we showed that Wee1 protein expression is cell cycle-regulated with protein accumulation in the G2/M phase. These data are in complete agreement with other reports that have monitored Wee1 protein expression during the cell cycle. In *S. pombe* Wee1+, transcripts did not fluctuate during the cell cycle, whereas the Wee1 protein underwent a moderate oscillation, being in S and G2 phases [52]. Furthermore, experiments following the behavior of the endogenous *S. cerevisiae* Swe1 protein concluded that Swe1 is stable during G2/M and not degraded until exit from mitosis [67,68]. The fact that the expression of TbWee1 is so strongly linked to the G2/M phase of trypanosomes and TbWee1 is expressed in the proliferative procyclic and bloodstream slender forms also fits well with a possible role for this protein kinase in cell division at G2/M. 

In this study, we showed that depletion of TbWee1 from the procyclic form of *T. brucei* produced a growth defect resulting in an enrichment of sub-G1-phase cells and a decrease in the percentages of the G2/M phase, which correlated with an increase in the number of slender zoids (0N1K) and abnormal (1N0K) cells, and a decrease in the number of 1N1K cells. This could be explained if cytokinesis was prematurely initiated following depletion of TbWee1, resulting in a 1N2K dividing cell giving abnormal daughter cells before it has the chance to go through mitosis. This could account for the lack of accumulation of 1N2K cells with a small decrease in the number of 2N2K cells and an increase in the number of abnormal cells when TbWee1 was depleted. 

Interestingly, knockdown of Wee1 by siRNA has been found to reduce viability of breast cancer cells but not of normal mammary epithelial cells [69]. Inhibition of Wee1 in cancer cells resulted in the accumulation of DNA damage, alteration in cell cycle regulation with an arrest in the S-phase of the cell cycle, increased sub-G1 DNA content, and induction of apoptosis [69]. It has been shown that cells with intact G1-checkpoint arrest, such as normal cells or cancer cells with intact p53 signaling are less dependent on the G2-checkpoint arrest and are, therefore, not as sensitive to Wee1 inhibition [70]. Furthermore, plants lacking a functional Wee1 are indistinguishable from wild-type plants when grown under non-stress conditions but are extremely sensitive to replication-inhibiting chemicals, showing a root growth inhibition phenotype [71]. Thus, although Wee1 might lack a function as a cell cycle regulator under non-stress conditions, its kinase activity seems to be essential upon replication stress [72]. 

In summary, we showed for the first time in trypanosomatids the presence of a protein that belongs to the Wee1 kinase family. We demonstrated that the parasite enzyme can rescue the wee phenotype of *S. pombe* mutants and that it exhibits tyrosine autophosphorylation activity. Although under the conditions used in this experiment the silencing of TbWee1 did not produce a strong phenotype, it led to a slower population growth rate and the appearance of a subG1 population.

Even though the specific function of TbWee1 is yet to be determined, we established the presence of this protein kinase in different stages of the parasites and in the G2/M phase of the cell cycle. Future research will focus not only on the identification of the substrates of TbWee1 but also on its specific localization, and we are currently engaged in this task.
